# First person – Amanda Miles

**DOI:** 10.1242/dmm.049372

**Published:** 2021-12-07

## Abstract

First Person is a series of interviews with the first authors of a selection of papers published in Disease Models & Mechanisms, helping early-career researchers promote themselves alongside their papers. Amanda Miles is first author on ‘ Usher syndrome type 1-associated gene, *pcdh15b*, is required for photoreceptor structural integrity in zebrafish’, published in DMM. Amanda is a PhD student in the lab of Vincent Tropepe at the University of Toronto, Toronto, Canada, investigating disease modelling for retinal development and disease mechanisms.


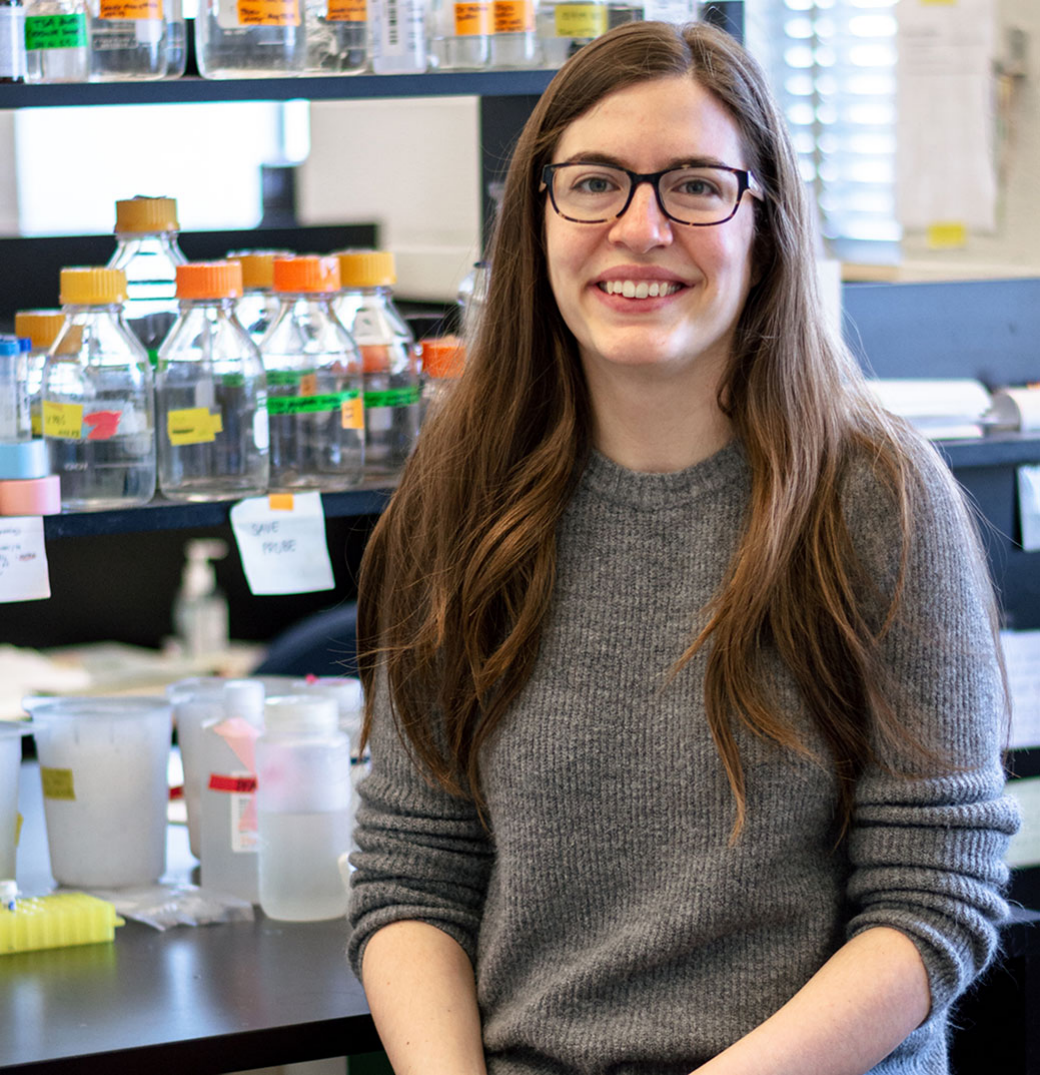


**Amanda Miles**

**How would you explain the main findings of your paper to non-scientific family and friends?**

In this paper, we studied a rare genetic disorder called Usher syndrome (USH), which is the leading inherited cause of combination deaf-blindness. We focused on the most severe form of this disorder, called USH type 1. Despite the amazing research on the ear defects associated with this disorder, the aetiology of blindness for USH type 1 has remained poorly understood, mainly because the animal mutant models (mice) used in research thus far only show defects in the ear and not in the eye/retina. Given that mouse models haven't been as useful in this field, our goal was to use zebrafish to generate a mutant model of a gene associated with USH type 1, called protocadherin-15, that could provide the community with a greater understanding of the defects that occur in the retina. Luckily, the zebrafish mutants we generated showed striking defects in the structure of the light-sensing cells of the retina, called photoreceptors. These structural defects occurred quite early in the development of photoreceptors, implying that this blinding disease may have a developmental origin rather than be a purely degenerative defect.

“The zebrafish mutants we generated showed striking defects in the structure of the light-sensing cells of the retina, called photoreceptors.”

**What are the potential implications of these results for your field of research?**

I hope our detailed analysis of the cellular defects in the retina in our zebrafish mutants can eventually inform the development of treatments for those with USH who are progressively losing their sight. What was really lacking in the field was a good animal model, and now that we have found and described the key defects in the retina associated with mutations in the USH gene protocadherin-15, one of our next goals is to understand how we can prevent these defects from happening. I believe our model opens the doors for far greater treatment-related research for USH than we had before.

**What are the main advantages and drawbacks of the model system you have used as it relates to the disease you are investigating?**

The organisation of the zebrafish retina and structure of photoreceptors is very similar to that of humans, making it a good model for the retinopathy of USH. Our study shows that zebrafish can model USH-associated retinopathies quite well and help inform us on defects that occur in the retina. As mentioned, this contrasts with previously described mouse models, which have shown inconsistent results and fail to exhibit clear retinal phenotypes. It's thought this is partially because mice photoreceptors lack structural features that are present in zebrafish and humans, called calyceal processes, where USH proteins are found to be expressed. Who would have thought zebrafish could be better than mice for modelling some human disorders?

A drawback for our model, however, is the duplication of genes in zebrafish, which commonly results in two or more paralogous genes. Protocadherin-15 exists in two genes in zebrafish, a *pcdh15a* and a *pcdh15b* gene, and, in this study, we only mutated *pcdh15b*. Although previous characterisation of *pcdh15a* mutants has not indicated retinal defects, the degree to which *pcdh15a* can function in the retina or mitigate the defects we observed is still not fully known.

**What has surprised you the most while conducting your research?**

Because of the progressive loss of photoreceptors in USH patients, most previous studies use cell death or thinning of the photoreceptor layer as an indicator of a retinal disease phenotype, but I was really surprised to see no indication of cell death or loss in the mutants we generated, at the time points analysed, and was initially discouraged by this. I then decided to look at the ultrastructure of the photoreceptors and that's when I noticed these abundant developmental structural abnormalities, even at a young age in zebrafish. This really changed our perspective on how USH develops and what cellular changes are going on before the clinical manifestation of photoreceptor cell death.

**Figure:**
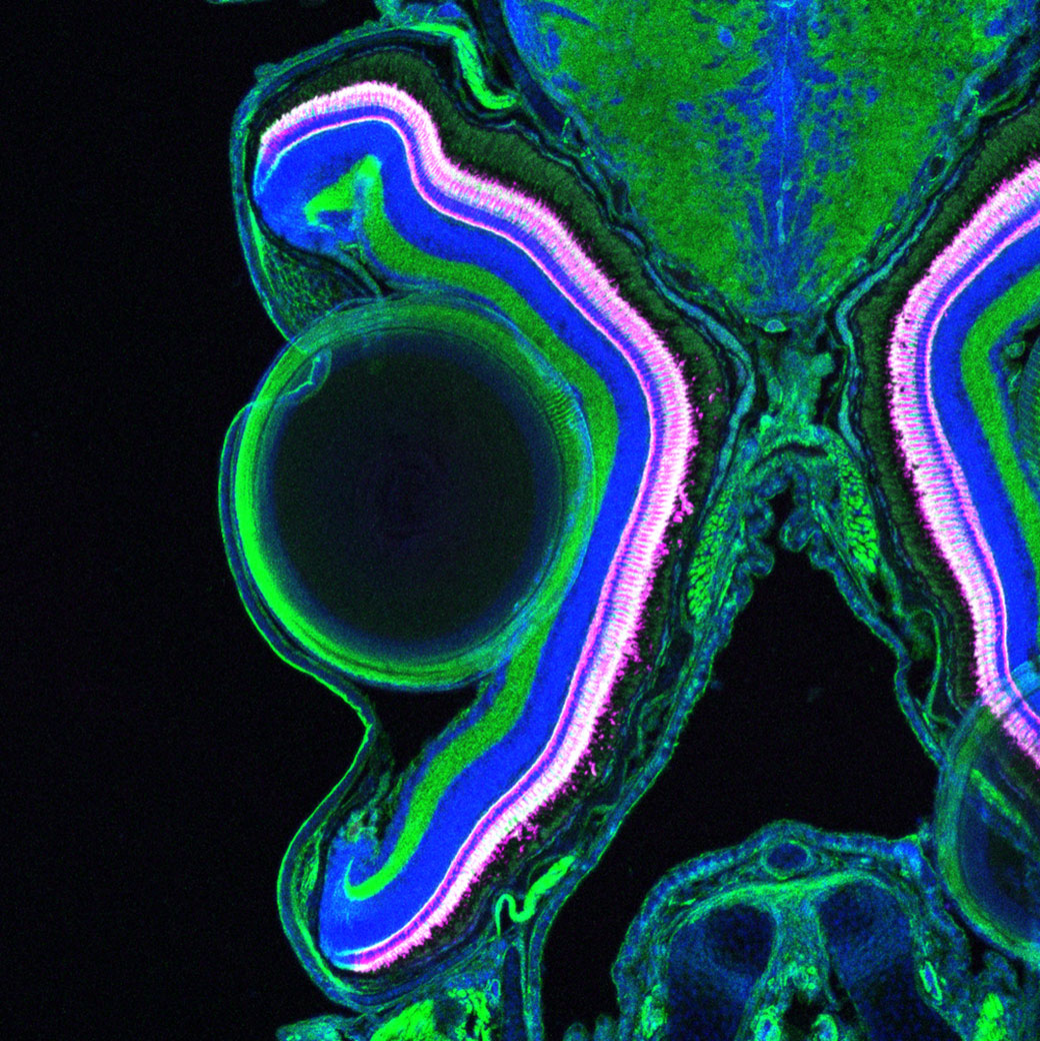
Coronal cross-section through the head and retina of a 1-month-old zebrafish. Blue labels cell nuclei (Hoechst), magenta labels cone photoreceptors (Zpr1) and green stains F-actin (phalloidin).

**Describe what you think is the most significant challenge impacting your research at this time and how will this be addressed over the next 10 years?**

There are many different isoforms for protocadherin-15, including alternative splice isoforms and protein isoforms, and it’s not well understood what all these different isoforms do in the retina or ear. It's been also suggested that certain animal models do not have clear retinal phenotypes because some isoforms may be partially retained. In terms of development of animal models and translation of this research to patients that have certain mutations, it drastically complicates our understanding of possible disease phenotype variability and limits our understanding of the disease progression. Key isoforms can start to be analysed through creation of different mutant models, to start to determine if there are key differences in function between them and how different mutations along the protein influence the phenotype. Great advances in CRISPR technology are really allowing us to influence the gene in different desired ways, and so I think creation of different variable mutant lines in zebrafish with different mutations, and/or different ‘rescue’ experiments with different isoforms in our current mutant, could lead to some interesting and insightful results about disease variability associated with different isoforms.

“During the pandemic, we saw a spike in miscommunication of science […]”

**What changes do you think could improve the professional lives of early-career scientists?**

Career-focused mentorship programs and science communication programs. The focus for scientists has largely been centred around research development – for technical skills, critical thinking and analysis. But it's just as important for scientists to develop tangible career goals and know how to navigate career options after their studies, whether inside or outside of academia. It's also just as important for scientists to be formally trained in science communication to share their research knowledge and the scientific process accurately to as many people as possible. During the pandemic, we saw a spike in miscommunication of science, and, as leading experts in our field, it should be our goal to share our research and its importance accurately. This not only informs the public but may lead to greater funding and importance placed on science programs, which help early-career scientists even more.

**What's next for you?**

Finishing and defending my PhD, by the end of the year. Because of my PhD work, I know that I would like to continue research focused on understanding the cell and molecular mechanisms that govern diseases, with the goal of these studies aiding in treatment developments. I'm looking to find the next position in my scientific career that aligns with these goals.
